# The State of Radiology Research in Ethiopia: A Scoping Review

**DOI:** 10.4314/ejhs.v34i1.9S

**Published:** 2024-10

**Authors:** Ashenafi Aberra Buser, Alemayehu Bedane, Kumlachew Abate Mekonen, Tesfaye Kebede, Shimels Hussien Mohammed

**Affiliations:** 1 Department of Radiology and Medical Radiologic Technology, School of Medicine, St. Paul's Hospital Millennium Medical College, Addis Ababa, Ethiopia; 2 Department of Radiology, School of Medicine, Addis Ababa University, Addis Ababa, Ethiopia; 3 Department of Public Health, School of Public Health, St. Paul's Hospital Millennium Medical College, Addis Ababa, Ethiopia

**Keywords:** Ethiopia, Radiology, Bibliometrics Analysis, Scoping Review

## Abstract

**Background:**

Radiology is an essential component of modern medicine and a rapidly evolving research field. The nature and dynamic of radiology research in Ethiopia remained largely unexplored This bibliometric scoping review was done to explore the current state of radiology research in Ethiopia.

**Methods:**

Literature search was conducted using PubMed, Scopus, Embase, Web of Science, and Google Scholar from inception to June 15, 2024. Study screening, review, and selection were performed using EndNote Reference Manager. The key indicators assessed include publication trends, research themes, publication utility, contribution and collaboration of individuals and institutions, and journal metrics. Statistical analysis was done using R and VOS viewer software.

**Results:**

Though low in volume, radiology research publication is increasing in Ethiopia, with 241 publications from 1968 to 2024, accounting for 0.03% of the global radiology research output. Top contributing institutions were Addis Ababa University, Jimma University, and St. Paul's Hospital Millennium Medical College. The studies were largely focused on case-reports, cross-sectional studies, and diagnostic imaging. Systematic reviews, meta-analyses, artificial intelligence and trials constituted only 3% of the studies. Most publications (96.7%) were done by academic institutions. Most frequently used journals were Ethiopian Medical Journal and Ethiopian Journal of Health Sciences, accounting for 29% and 15% of the total publications, respectively. Publications in Q1 journals was 12%. International collaboration was 7%, with the USA being the primary collaborator.

**Conclusion:**

To elevate the quality and impact of radiology research in Ethiopia, it is crucial to adopt contemporary and robust research methodologies, align research topics with global trends and technological advancements, and foster enhanced collaboration and productivity within the research community.

## Introduction

Radiology is an essential component of modern medicine, playing a crucial role in researching, diagnosing, and managing diseases (1, 2). The last two decades have seen a significant increment in radiology research activity and publications (3–6). The period has also seen a major rise in the themes, scopes, and complexity of radiology research designs, sampling methods, data size and analysis methods. This growth in robustness and productivity was in part driven by the availability of big datasets, advancements in statistical analysis software and imaging technologies such as magnetic resonance imaging (MRI) and computed tomography (CT) (2–6). Thousands of radiology scholarly outputs are being published every year. Most of the publications are made by the developed countries. The most contributing countries to global radiology research are United States of America, Europe, and Japan (7).

Radiology research is not limited to academia, with direct applicability to real-world medical practice by enhancing the diagnosis, management, and monitoring of diseases (5,6). Various medical devices and software are being innovated through radiology studies leading to quicker, safer, and more efficient imaging methods. Technological advancements are key to progress in radiology research; a new imaging modality or a major improvement on existing technologies often leads to new research directions that make this field dynamic and ever-evolving (1,2). Radiological findings are typically published in peer-reviewed journals but specialized radiology journals and other medical publications serve as main outlets. The themes of research conducted in the area of radiology have evolved over time. While earlier investigations focused on technicalities of imaging procedures, modern-day studies concentrate on image analysis, artificial intelligence applications and optimization of imaging protocols for specific clinical scenarios which show that the focus has shifted from obtaining clinically relevant information towards improved patient outcomes (3–6).

A scoping review is a preferred review methodology to systematically map and characterize a research field (8). The traditional systematic reviews and meta-analysis methodology is aimed to address specific research questions and uses narrow literature searching, screening, and selection criteria. On the contrary, the scoping review methodology employs wider and sensitive searching, screening and selection processes and criteria (1,9–13). This approach enables exploration of all relevant scholarly literature in a field to holistically map the overall landscape of research in the field by exploring key research themes, research volume, trends and intensity, and gaps in knowledge. Due to these reasons, the methodology has recently gained an increasing recognition by both scholars and policymakers in various health fields (14,15).

Despite the global advancements in radiology research, there is a significant knowledge gap regarding the state of this field in Ethiopia. Information on the volume, trend, focus, collaboration patterns, contributors and impact of radiology research conducted in the country is sparse. The lack of evidence impedes informed decision-making regarding research priorities, resource allocation, and capacity-building initiatives. To address this gap and gain a deeper understanding of the current landscape of medical radiology research in Ethiopia, a bibliometric scoping review analysis is particularly well-suited. Thus, this bibliometric scoping review was designed and conducted with the main aim of establishing the state of medical radiology research conducted in Ethiopia from inception to 2024. To this end, the study aimed to identify publication trends, key research themes, publication outlets, publication utility, journal metrics, contributions by institutions and authors, and collaboration patterns between authors, institutions and countries, and knowledge gaps and future research priorities in Ethiopian medical radiology. The findings from this scoping review will be instrumental in informing stakeholders to develop targeted strategies to enhance radiology research in Ethiopia.

## Materials and Methods

This review was conducted following the recommendations of the Meta-analysis of Observational Studies in Epidemiology (MOOSE) (16) and bibliometric scoping review guidelines (8), and reported following the Preferred Reporting Items for Systematic Reviews and Meta-Analyses (PRISMA) guidelines (17).

**Literature search**: A comprehensive literature search was conducted to identify studies related to medical radiology research in Ethiopia across five databases: PubMed, Scopus, Embase, Web of Science, and Google Scholar. To ensure exhaustive coverage, the search included a combination of keywords and Medical Subject Headings (MeSH) terms such as “radiology”, “medical radiology” “medical imaging”, and “Ethiopia”. Boolean operators (AND, OR) were utilized to combine search terms. Truncation and wildcards were also employed to capture variations of search terms. The literature search was limited to articles published in English. The systematic database search included studies published from inception to June 15, 2024. The search was done across all databases during the period June 11-15, 2024. Additionally, the reference lists of relevant articles were checked to identify any studies that might have been missed in the initial database search. Grey literature was also checked by searching institutional repositories in the field. The database-specific search models were the following: PubMed (“radiology” [MeSH Terms] OR “radiography” [MeSH Terms] OR “radiography” [MeSH Terms] OR “radiology” [Title/Abstract] OR “imaging” [Title/Abstract] OR “radiography” [Title/Abstract]) AND “humans” [MeSH Terms] AND ((“Ethiopia” [MeSH Terms] OR “Ethiopia” [Title/Abstract]) AND “humans” [MeSH Terms]) AND “English” [Language]; Scopus (TITLE-ABS-KEY (imaging AND Ethiopia)) OR (TITLE-ABS-KEY (radiology AND Ethiopia)) AND (LIMIT-TO (LANGUAGE, “English”)) AND (LIMIT-TO (EXACTKEYWORD, “Human”)); Embase (‘radiology Ethiopia’ OR ((‘radiology’/exp OR radiology) AND (‘Ethiopia’/exp OR Ethiopia)) OR (imaging AND Ethiopia)) AND ‘human’/de AND [English]/lim; Google Scholar “radiology” OR “imaging” AND Ethiopia.

**Eligibility criteria**- Studies were included if they met the following criteria:

- Focused on medical radiology in Ethiopia using different approaches;
- Used radiological tools and procedures as the main measurement, diagnostic, and analysis approaches;
- Published in a peer-reviewed journal;
- Case reports, case series, editorials, and empirical research articles, including observational studies, experimental studies, qualitative research, systematic reviews, and meta-analyses;
- Published from inception to June 15, 2024;
- Available in full text and written in English.

Exclusion criteria were:

- Studies not primarily focused on Ethiopia;
- Studies done on non-human subjects;
- Letters and commentaries;
- Studies published in predator journals;
- Under review (unpublished) studies;
- Articles not available in full text or not in English;
- Conference papers, conference reviews, books, book chapters, notes, erratum, etc.

**Study screening**: The study screening process was conducted in two stages to ensure thorough and unbiased selection. In the first stage, titles and abstracts of all identified articles were screened to assess relevance based on the eligibility criteria. In the second stage, the full texts of potentially eligible articles were retrieved and reviewed to confirm eligibility. Articles that did not meet the inclusion criteria upon full-text review were excluded, and reasons for exclusion were documented. The screening process was done using EndNote Version 21 and Mendeley reference managers. The study screening was performed independently by two researchers, ABW and SHM. Any inconsistencies were resolved through joint discussion.

**Data extraction**: Data were extracted from the included studies using a standardized data extraction Excel sheet specifically designed for this review. To enhance the reliability of the data extraction process, pilot testing of the data extraction form was conducted on a sample of studies to refine and standardize the process. The data extraction was performed independently by two researchers, ABW and SHM. Any inconsistencies were resolved through joint discussion. The following variables were collected from each included study in this work:

- Author(s) name;
- Title of studies;
- Year of publication;
- Journal of publication: Journal title, type, impact factor, quartile (Q1, Q2, Q3, Q4), volume, indexing, and audience.

**Citations**; The number of times the article is cited by other scholarly works;

**References**: The number of scholarly works included in the report;

**Study place and setting**: The time and place where the study was conducted.

**Characteristics of studies**: study type, study design, thematic area, and tools used.

**Authorship metrics**: number of radiology-specific publication outputs and citations acquired, overall H-index, international collaboration, co-authors' name, co-authors' number, percentage of publication in Q1 journals, and share in top 25% cited articles (18,19).

Affiliation metrics: authors' country and institutional affiliations (18,19).

**Description of main variables**: Publication output (number of publications): This is the total number of research articles, books, or other scholarly works a researcher has published.

**Citations**: This refers to the number of times a researcher's work has been referenced by other scholars in their publications. Higher citations indicate greater impact and influence in the field (18,19).

**H-Index**: This is a composite metric that considers both productivity and citation impact. A researcher with an h-index of h has published h papers, each of which has been cited at least h times (20).

**International collaboration**: This refers to whether a researcher has collaborated with colleagues from other countries and can bring together different expertise and resources.

**Percent of publication in Q1 journal ranks**: Journals are often ranked by their impact factor, which is a measure of how often the articles published in that journal are cited. Q1 represents the top quartile (25%) of journals in a field, so this metric shows the percentage of a researcher's publications that appeared in the most prestigious journals (19).

**Percent of publications in the top 25% of most cited documents**: This metric shows what percentage of a researcher's publications are among the most highly cited documents in their field globally.

**Citation**: A link signifies that one publication cites another.

**Bibliographic coupling**: This link connects publications that share a high number of citations from other publications. The distance between linked publications can indicate the thematic similarity between their research areas (14,21).

**Authors' collaboration (Co-authorship)**: Collaboration between two or more authors on a research publication (18).

**Country collaboration**: Analysis of research collaborations between authors affiliated with different countries (14,21).

**Thematic research analyses**: Identification of research areas where different studies converge based on the co-occurrence of thematic keywords or phrases (14,21).

**Top cited articles**: This refers to the publications that have garnered the highest number of citations from other scholarly works. These articles are considered highly influential and have significantly impacted the field (18,19).

**Top cited authors**: Researchers in Ethiopian radiology whose publications have been cited most frequently by other scholars. This recognition indicates their significant contributions to the field and the widespread recognition of their work (15).

**Open Access Journals (Gold)**: These journals make all their articles freely available online immediately upon publication. No subscription fees are required to access the research.

Gold Open-Access with Author Processing Charges (APCs): While freely accessible to readers, authors may incur publication fees to cover the journal's operational costs (15).

Hybrid Open-Access Journals: These journals publish a mix of open-access and subscription-based articles. Some articles may require an access fee, while others are freely available (15).

Subscription-Based Journals (Bronze): Access to the full text of articles in these journals typically requires a paid subscription, either by individual readers or institutions (15).

Review Articles: These articles synthesize and critically evaluate existing research on a specific topic (22).

**Research articles**: These articles report the findings of original research studies conducted by the authors. They include clinical trials, observational studies, and laboratory experiments (22).

**Case reports**: These reports document a single, unique, or interesting patient case that may offer valuable insights into specific diseases or treatment approaches (22).

Editorials: These are perspective pieces or viewpoints written by editors or invited experts who comment on current issues, emerging research findings, or controversies (22).

**Statistical analysis**: The characteristics of the included studies were summarized using descriptive statistics. A bibliometric analysis was conducted to identify publication trends, key research themes, and prominent studies, journals, researchers, and institutions. The annual growth of published documents served as an indicator of radiology research productivity in Ethiopia. The studies and their extracted data were exported from MS Excel spreadsheets to R Studio for further analysis. VOSviewer software was used for constructing and visualizing bibliometric networks, links, and maps (21).

The impact of each included study was assessed, with the top 15 most impactful articles identified and presented in this report based on their citation counts (in descending order). The ranking and contribution of institutions to Ethiopian radiology research were determined by the authors' affiliations mentioned in the assessed documents. Additionally, influential journals and researchers were identified. The ranking of publication journals was determined by the percentage of publications appearing in the specific journals.

Authors' contribution was determined by research output, citations, H-index, international collaboration, publication in Q1 journals, and the percentage of their work within the top 25% of most cited articles. A Standard Competition Ranking (SCR) using the Scopus ranking system was developed based on research output, citations, international collaboration, and the share of their publications in Q1 journals and the top 25% cited documents in the radiology field. The normalization process accounts for potential variations in these metrics, leading to a more robust and comprehensive evaluation of author contribution. Weights were assigned to each metric based on Scopus recommendations (23): 0.3 for research output, 0.3 for citations, 0.2 for publication in Q1 journals, 0.1 for international collaboration, and 0.1 for publication in the top 25% cited documents. Only radiology-specific research outputs and citations were considered when determining research output and citations. H-index of authors was obtained from Google Scholar or Scopus if unavailable in Google Scholar. However, H-index was excluded from the author's normalized relative ranking index because not all authors' publications were radiology-specific, and H-index does not differentiate radiology-specific research impact (20).

Additional bibliometric analyses were done to identify the dominant research themes, productive scholars, and collaborative fronts between authors and countries. The minimum thresholds for inclusion in the collaboration and network analyses of authors, countries, and research thematic areas were 5, 2, and 5 articles, respectively. To this end, the following three VOSviewer analyses were conducted (14, 21). First, networking analysis was done by running the RIS data in VOSViewer software. This enabled the conversion of the bibliometric data into a visual map where authors, institutions, countries, and research themes acted as nodes connected by links based on the nodes' collaboration, co-occurrence, or co-authorship. The size of a node reflects its relative prominence or weight in the network. The thickness of a link indicates the strength of the relationship between the nodes. Though not always true due to other nodal influences, the distance between nodes generally signifies the relative connectedness or strength of correlation between the items connected. Finally, density analysis was conducted to identify prominent collaboration areas and validate if they correspond to the findings of the networking analyses, for example, to check whether the most productive researchers and research themes are also the main collaboration fronts. Denser regions represent more engaged scholars, prominent research fields, and collaboration fronts. Then overlay analysis was done to compare publication networks from different periods to identify emerging or declining research themes, authors, and collaborations, highlighting the evolution of the Ethiopian radiology research landscape overtime (21).

## Results

**Literature search result and characteristics of included studies**: A total of 2054 documents were found by searching the five databases, repositories and the reference list of the top 15 most cited articles. After screening the title and abstract of the retrieved studies, 432 reports were found eligible for full-text review. Full-text reviewing of the 432 studies resulted in 241 eligible studies. The full list of the 241 reports included in this work is provided as a supplementary file (Supplementary File 1).

Table 1 shows a summary of the main characteristics of the radiology research conducted in Ethiopia and published until 2024. Overall, the studies gained a total of 1872 citations, translating to an average citation of 4.7 per paper per year. The publications used an average of 17 references and 4 authors per paper. A total of 151 authors were identified from 76 institutions and 26 countries. The majority of the authors were affiliated with academia (96). Over two-thirds of publications were published in fully accessible open journals. The publications encompassed various report types, including 240 articles, 2 editorials, 3 reviews, and 4 longitudinal studies.

**Table 1:** Summary of characteristic of included studies

Domain	Metric		No. or %
Publication	Number of papers published		241
	Publication year		1968-2024
	Publication per year		4.3
Citation	Total citations		1872
	Citation years		56
	Average citation/year		33.4
	Average citation/paper		7.8
Reference	Total references		4246
	Average references/paper		17.6
Author	Total authors		151
	Average	authors/paper	4
	(mean)		
	Average	authors/paper	3
	(median)		
	Average (mode)	authors/paper	2
	Average citation per author		12.4
Affiliation	Total affiliations		76
	Affiliations (countries)		26
	Affiliations (only academic)		219
	Affiliations (non-academic)		8
	Affiliations (mainly-academic)		22
Journal access	Gold		226
	Hybrid gold		10
	Bronze		5
	Article		232
	Editorials		2
	Review		3
	Longitudinal		4
	Qualitative		2
Publication journal	Publications in Q1 Journals		11.8%
	Publications in Q2 Journals		14.2%
	Publications in Q3 Journals		25.7%
	Publications in Q4 Journals		35.6%
	Publications in Q1 citations		0.32%

**Figure 1:**
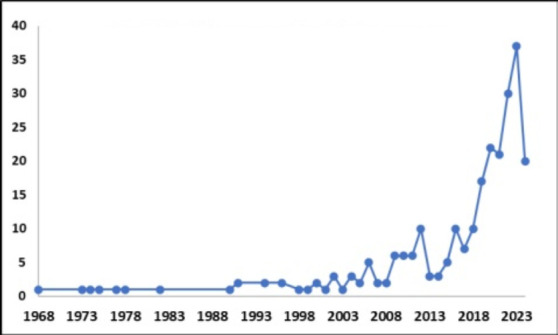
Trend of Radiology Research in Ethiopia, 1968-2024

**Trend of radiology research publication in Ethiopia**: In terms of the trend of publications, there was no major change in the volume of publications until 2000. However, the period after 2000 has seen a significant and consistent increment in the volume of publications, reaching a peak in 2023. The trend as depicted in Figure 1 suggests the growth would continue in the coming years.

**Top most cited radiology scholarly publications in Ethiopia**: Table 2 presents the 15 most cited scholarly works published between 1968 and 2024 in the radiology field in Ethiopia. In total, the 15 articles gained 830 citations, accounting for a significant portion (44.3%) of the total citations of the 241 Ethiopian radiology research works (830 out of the total 1872). The most cited work, by Ayana G. et al (24), acquired 122 citations by June 15, 2024. It was a review paper published in 2021 and focused on transferring learning in breast cancer diagnoses via ultrasound imaging.

**Table 2:** Top 15 most cited radiology articles from Ethiopia, 1968-2024

Authors	Title	Year	Cites	Type	Rank
Ayana G.	Transfer learning in breast cancer diagnoses via ultrasound imaging	2021	122	Review	1
Aderaye G.	The relationship between disease pattern and disease burden by chest radiography, M. tuberculosis load, and HIV status in patients with pulmonary tuberculosis in Addis Ababa	2004	96	Article	2
VD Heuvel	Automated Fetal Head Detection and Circumference Estimation from Free-Hand Ultrasound Sweeps Using Deep Learning in Resource-Limited Countries (26)	2019	82	Article	3
Yadeta D.	Prevalence of rheumatic heart disease among school children in Ethiopia: A multisite echocardiography-based screening	2016	81	Article	4
Aderaye G.	Pneumocystis jiroveci pneumonia and other pulmonary infections in TB smear-negative HIV-positive patients with atypical chest X-ray in Ethiopia	2007	54	Article	5
Tessema T.A.	An evaluation of the diagnostic value of clinical and radiological manifestations in patients attending the Addis Ababa tuberculosis centre	2001	49	Article	6
Geleto G.	Mean Normal Portal Vein Diameter Using Sonography among Clients Coming to Radiology Department of Jimma University Hospital, Southwest Ethiopia	2016	48	Article	7
Hawaz Y	Ultrasound assessment of normal portal vein diameter in Ethiopians done at Tikur Anbessa Specialized Hospital	2012	45	Article	8
Mor Z.	Chest radiography validity in screening pulmonary tuberculosis in immigrants from a high-burden country	2012	44	Article	9
Sorri G.	Patterns of neural tube defects at two teaching hospitals in addis ababa, ethiopia a three years retrospective study	2015	40	Article	10
Admassie D.	Incidence of normal pineal and chroids plexus calcification on Brain CT (computerized Tomography) at Tikur Anbessa Teaching Hospital Addis Ababa, Ethiopia	2009	39	Article	11
Tessema T.A.	Clinical and radiological features in relation to urinary excretion of lipoarabinomannan in Ethiopian tuberculosis patients	2002	38	Article	12
Berhe N.	Large scale evaluation of WHO's ultrasonographic staging system of schistosomal periportal fibrosis in Ethiopia	2006	31	Article	13
Wakjira E.	Implementing ultrasound-guided hydrostatic reduction of intussusception in a low-resource country in Sub-Saharan Africa: our initial experience in Ethiopia	2018	31	Article	14
Gleason R.L.	A safe, low-cost, easy-to-use 3D camera platform to assess risk of obstructed labor due to cephalopelvic disproportion	2018	30	Article	15

**Figure 2:**
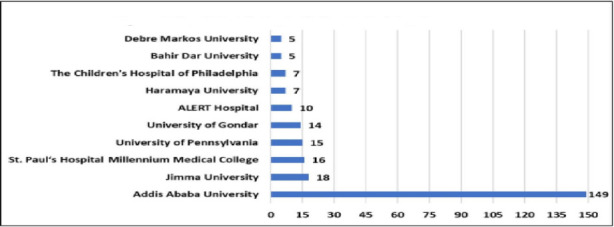
Top 10 Contributing Institutions to Radiology Research in Ethiopia, 1968-2024

**Institutional contribution to radiology research in Ethiopia**: This review showed the involvement of various national and international organizations involved in radiology research in Ethiopia. Academic institutions were relatively more productive in publishing than private and nongovernmental organizations. The non-academia sector contributed only to 3.3% of the total radiology research in Ethiopia. Figure 2 depicts the top 10 most contributing institutions to radiology research in Ethiopia during the period 1968-2024. Addis Ababa University was the top contributor with 149 contributions accounting for 60% of the total research output during this period, followed by Jima University with 18 contributions (7%). St. Paul's Hospital Millennium Medical College contributed 16 publications, standing third nationally with a 6% share of the national radiology research output in Ethiopia.

**Journals of publications of radiology research in Ethiopia**: The main academic journals that published most of the Ethiopian radiology scholarly were explored. The leading publishing journal during the whole period was Ethiopian Medical Journal, publishing 72 articles and accounting for 29% of the total publications. The second leading publishing journal was Ethiopian Journal of Health Sciences, publishing 38 articles (15% share of publication). Together, these two Ethiopian journals published 110 articles, accounting for 44% of the total publications. Journal life-year adjusted comparison of the performance of the two journals showed that Ethiopian Journal of Health Sciences was more productive and preferred among radiology scholars engaged in radiology research in Ethiopia.

**Prominent radiology researchers in Ethiopia**: Table 3 presents the list, scholarly contributions, and relative ranking of the most influential scholars in the field of radiology in Ethiopia. Tesfaye Kebede was the most prominent scholar in the field by total research outputs, contributing 39 publications or 16% of the total publications. Seife Teferi was the most cited scholar, garnering 241 citations or 2% of total citations in the field. Getachew Assefa stood as the top-ranked radiology scholar in Ethiopia based on the overall standard competition ranking (SCR). The list of other prominent scholars, their contributions to the field, and information about other authorship metrics like H-index, authorship coloration, and publication in Q1 are shown in Table-3.

**Table 3:** Top 10 scholars contributing to radiology research in Ethiopia, 1968-2024

Scholar Name	Affiliation	Output	Cites	H Index	Collaboration^*^(%)	Q1J^†^(%)	Q1P^‡^(%)	SCR^§^	Net Rank
Getachew	Addis Ababa								
Assefa	University	22	110	12	47.4	47.4	42.1	0.673559	1
Seife	Addis Ababa								
Teferi	University	19	241	8	25	8.3	8.3	0.540718	2
Tesfaye	Addis Ababa								
Kebede	University	39	158	6	17.4	0	4.3	0.535948	3
Assefa	Addis Ababa								
Getachew	University	5	88	12	20	44.4	40	0.452459	4
Kassa									
Darge	Penn medicine	6	37	44	38.3	43.5	42.9	0.430582	5
Melkamu	Jimma								
Berhane	University	7	39	11	59.5	44.4	21.6	0.430344	6
Daniel	Addis Ababa								
Zewdeneh	University	17	68	3	25	25	0	0.357433	7
Daniel	Addis Ababa								
Admassie	University	30	80	5	0	0	0	0.330354	8
Yocabel	Addis Ababa								
Gorfu	University	11	55	5	50	16.7	0	0.303914	9
Abebe	Debremarkos								
Habtamu	University	7	34	4	25	25	16.7	0.277114	10
Elias Kadir	Jimma Univerity Addis Ababa	5	5	2	0	50	0	0.244686	11
Asfaw	University	13	65	4	33.3	0	0	0.236879	12
Mesfin	Jimma								
Zewdu	University	5	25	4	0	33.3	14.3	0.236115	13
Yonathan Gebrewold Alemayehu	University of Gondar	5	29	3	16.7	16.7	16.7	0.208356	14
Bedane	SPHMMC	6	6	3	33.3	0	0	0.109589	15

* International collaboration level

† Share of publication Q1 journals

‡ Share of publication in Q1 citations

§ Standard Competition Ranking (SCR)

**Thematic focus of radiology research in Ethiopia**: Figure 3A presents the results of the focus of the published radiology studies. The network visualization indicates a central role for “retrospective studies” and article” themes, signifying their frequent occurrence in radiology research. Additionally, thematic clusters emerge, highlighting closely related research areas. The blue cluster focuses on “retrospective studies” and “magnetic resonance imaging,” while the green cluster encompasses “cross-sectional studies,” “ultrasonography,” “reference values,” and “education.” finally, the red cluster groups “article,” “radiography,” “echography,” and “computer-assisted tomography.” “radiography” and “ultrasound” were connected to various themes across clusters, suggesting their broad applicability within radiology research. The density map corroborated the network visualization's findings by pinpointing key thematic areas like “retrospective studies” and “article” as central research fronts. The density map also revealed themes like “education”, “MRI” and “predictive value of tests” as less explored fronts, suggesting potential areas for increased exploration within Ethiopian radiology research.

**Figure 3 A:**
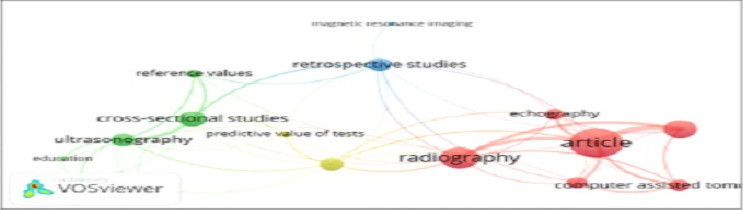
Main thematic areas of radiology research in Ethiopia

**Authors' collaboration in radiology research in Ethiopia**: Figure 3B visualizes the co-authorship pattern among researchers involved in radiology research in Ethiopia. Each node represents an author, with the size of the node indicating the prominence of the author in the network. Based on the two visuals in Figure-3B and Figures-3C, the most prominently and actively collaborating author was Tesfaye Kebede followed by Daniel Admassie, Getachew Assefa, and Seife Teferi, who all appear as larger nodes, indicating their significant contributions in the field. Other notable authors such as Gorfu, Darge, and Abebe are also connected, showing a collaborative network among these researchers and the interlinked nature of radiology research in Ethiopia. The graph also indicates a clustered pattern of collaboration among authors, with different colors representing groups of closely working authors. For example, Tesfaye Kebede, Daniel Admassie, and Getachew Assefa form central nodes in their respective clusters, connected to multiple other researchers within and across clusters of collaborations.

**Figure 3 B:**
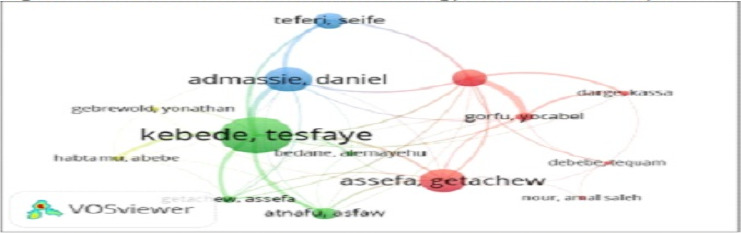
Authors collaboration for radiology research in Ethiopia

**Figure 3 C:**
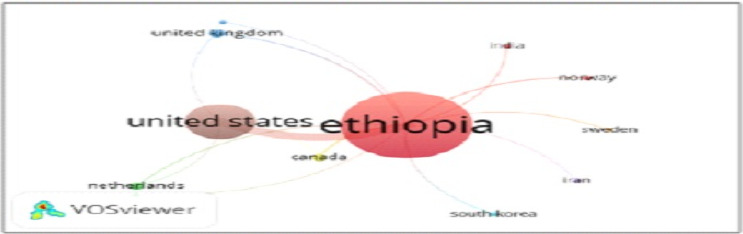
Countries collaboration for radiology research in Ethiopia

**Country collaboration for radiology research in Ethiopia**: Figure 3C depicts the pattern of collaboration among countries for radiology research in Ethiopia. The main partnering countries were USA, followed by UK and Canada. These countries form the primary nodes around Ethiopia, indicating better research ties. Other countries such as the Netherlands, India, Norway, Sweden, Iran, and South Korea also maintained collaborative links with Ethiopia, though these connections were less prominent compared to the three primary partners. The density visualization further underscored the relatively stronger collaboration between Ethiopia and USA.

## Discussion

This bibliometric scoping review systematically analyzed the state of radiology research in Ethiopia. It quantitatively assessed the trend, thematic areas, collaboration, productivity, publication outlets, and impact of radiology research conducted in Ethiopia. The study provided comprehensive and important insights into the nature of the field. The period after 2000 has seen a significant and consistent rise in the publication of radiology research works in Ethiopia. This finding reflected positively on the growth and prospect research in the field and was consistent with the report of various global studies which also showed a recent research surge in the field (7,24). The recent increment in radiology research in Ethiopia could be attributable to, but not limited to, the expansion of radiology schools in the country as well as the global technological advance in radiology, computing powers, and statistical analysis software (2,7).

Despite the historical rise in radiology research outs in Ethiopia in the last 2 decades, the overall volume was significantly low compared to the expected level and trend in other nations. Scopus alone, a major scientific database, had indexed over 747,621 articles related to radiology, nuclear medicine, and imaging by June 15, 2024. Ethiopia's contribution to this research pool was only 241 or 0.03%. Given Ethiopia's population size and the burden of disease it faces, one might expect a more substantial contribution to global radiology research. When adjusted for population size, the above figures indicated that Ethiopia's contribution to global radiology research was 54 times lower than the expected level. This study also showed that Ethiopia's average radiology research publication rate was only 4.4 articles per year. This was significantly lower than the average number of publications in other countries (7,25). Countries like the USA, Japan, Netherlands, and Korea each produce thousands of radiology research papers annually, demonstrating the vast difference compared to Ethiopia's contribution (2,7). This disparity underscored the major under-representation of Ethiopian radiology research in the global context and the need for enhanced research efforts.

The thematic scope analysis revealed that Ethiopian radiology research was dominated by traditional and low-rigor research works such as case reports, descriptive studies, and cross-sectional designs. This finding was not aligned with the contemporary global radiology research themes like meta-analyses, machine learning, bid-data, advanced technology and AI utilization (26–29). The lack of high rigour studies could be due to low research capacity, low research budget, low emphasis and attention to research as well as poor access to advanced statistical analyses software in Ethiopia. The study topics, target community, and publication outlets were also mostly confined to Ethiopia. This local orientation has its own advantages in focusing on country-specific health issues and promoting the growth of national journals. On the contrary, it might have limited the international utility and influence of Ethiopian radiology research on the global stage. The low citation rates of the articles, 5.5 citations per article, could be partly attributed to publishing more in local journals with lower quartile and impact factors. Only 12% of Ethiopian radiology publications appeared in top-tier international journals, hindering their visibility and perceived quality. This was evidenced by the findings of this work that Ethiopia-originated radiology scholarly works accounted for only 0.3% of the 25% globally most cited articles in the field. A balanced utilization of both international and national journals might help to achieve both aims. Not only the journals, but the largely empirical nature of the studies might have also contributed to the low influence and utility of publications from Ethiopia. Evidence shows secondary studies like reviews and meta-analyses are more citable, utilized, and influential (22,26–29). In agreement with this, this study also found that the most cited articles in Ethiopian radiology research were also review ones.

Only a few institutions in Ethiopian radiology had high levels of research productivity. Addis Ababa University, Jimma University and St. Paul's Hospital Millennium Medical College were the major ones. Non-academic institutions' research engagement and contribution to radiology research were almost none in Ethiopia. This could be partly due to the private sector in Ethiopia prioritizing only financially profitable imaging services over research. It could also be due to a lack of awareness about the financial potential of research-oriented radiology, such as establishing and providing big data and imaging centers to large companies for machine learning, algorithm development, and AI research. On the other hand, there was a small number of productive researchers accounting for most of the publications made in the radiology field in the country. This low number of researchers in the field might be suggestive of a lack of interest, capability, and involvement in research at large among both academicians and clinicians, but more particularly among clinicians. Ethiopian radiology researchers produced fewer papers as compared to their global peers and received fewer citations and international collaborations. This underscored the need for more local and international partnerships to improve the productivity of both individual researchers and institutions in scientific work in Ethiopia (7,23,24,25).

This study indicated that Ethiopian radiology research required efforts to grow further to bring it up to global standards. Enhancing both the quantity and quality of research output is important. Focusing on robust designs such as systematic reviews, meta-analyses, and longitudinal and randomized controlled trials is needed to improve research quality and impact (26–29). Aligning research topics with global trends in artificial intelligence, big data, and advanced imaging technologies is also essential (1,2). Moreover, publishing in high-impact journals and fostering international collaboration will enhance visibility and influence (24). This requires creating research infrastructure, budget, capacity-enhancing training and motivating incentives for research as well as raising awareness about the importance of research and publication for professional career and advancement of the field. Radiology research in Ethiopia stands to benefit significantly from increased international collaboration, particularly in high-tech areas like AI and machine learning. By partnering with global institutions, Ethiopia can access advanced technologies and expertise, enhancing both the volume and quality of research. Collaborative efforts could focus on developing AI tools for diagnostic imaging, which would be particularly valuable in resource-limited settings. Furthermore, these partnerships can facilitate training programs and exchange initiatives, empowering Ethiopian researchers with cutting-edge skills. Ultimately, such collaborations could lead to innovations that improve healthcare outcomes across the region. However, further in-depth explorations are needed to understand the barriers and enablers of integrating research into Ethiopian radiology practice.

This study had some strengths and limitations worth mentioning. First, the study was the first bibliometric scoping review on the field of radiology research in Ethiopia. Second, the study followed a comprehensive review approach, searching multiple databases and including a large body of literature. Third, the utilization of bibliometric visualization tools like R Studio and VOS Viewer software enabled to gain quantitative insights into the research productivity and collaboration patterns. One of the limitations of the study was its reliance on available databases and the likelihood of excluding relevant studies published in unindexed and predatory journals. The use of only published works would have also undermined the research work for not all research works are published. Additionally, the primary reliance on thematic analysis, citation, and journal metrics for assessing research quality and impact might not fully reflect the quality of radiology research in Ethiopia. The search was restricted to the English language and it might have excluded relevant studies published in other languages.

In conclusion, this bibliometric scoping review identified a positive trend in the volume of Ethiopian radiology research over the past two decades. However, by most global standards, the quality and quantity of this research output remain suboptimal. The thematic focus on traditional and methodologically weaker designs, publication in local journals, and limited collaborative efforts (both national and international) had likely restricted the impact and visibility of Ethiopian radiology research. To foster research in this field, it is necessary to establish research infrastructure, allocate adequate budgets, provide capacity-building training programs, and offer motivating incentives for researchers. Additionally, raising awareness about the significance of research and publication for professional growth and advancement within the field is crucial. This, in turn, would contribute to the development of a more robust and impactful research landscape, ultimately benefitting patient care, radiology education, and the broader Ethiopian healthcare system.
